# A comparative study of antimicrobial prescribing practices for common infectious syndromes among physicians and nurse practitioners in a safety-net hospital

**DOI:** 10.1017/ash.2025.10058

**Published:** 2025-06-30

**Authors:** Aakash Balaji, Jessica Hua, Ben Pomerantz, Alfredo J. Mena Lora

**Affiliations:** 1 University of Illinois at Chicago, Chicago, IL, USA; 2 Saint Anthony Hospital, Chicago, IL, USA

## Abstract

Antimicrobial prescribing differences between physicians and nurse practitioners (NPs) remain poorly characterized. We compared prescribing practices at a safety-net hospital. NPs adhered more to pneumonia guidelines, while physicians had better adherence for abdominal and urinary infections. Ineffective therapy was more common for NPs. These gaps highlight important stewardship opportunities.

## Background

Optimizing antimicrobial use (AU) is essential in combating antimicrobial resistance (AMR).^1^ Studies estimate that up to 50% of prescribed antimicrobials may be unnecessary.^1^ Antimicrobial stewardship programs (ASPs) help improve AU by guiding appropriate antimicrobial selection and duration, leading to lower AMR rates, *C difficile* infections, and cost savings.^2^ Nurse practitioners (NPs) play an increasing role in inpatient care, particularly in smaller hospitals.^3^ Studies show NPs improve efficiency, enhance patient outcomes, and reduce costs.^4^ However, despite their growing presence, NP antimicrobial prescribing practices in inpatient settings remain poorly characterized. While studies in outpatient settings have shown higher inappropriate prescribing rates among NPs for upper respiratory infections compared to physicians, there is a paucity of data on NP prescribing practices for common infectious syndromes in hospitalized patients.^5,6^

Small hospitals are a cornerstone for healthcare delivery in the United States, where over two thirds of hospitals have fewer than 200 beds and 10% fewer than 25.^7^ Larger hospitals are more likely to have robust ASPs, while smaller hospitals often lack ID-trained pharmacists or multidisciplinary stewardship teams.^8^ Given the growing role of NPs across the United States, particularly in smaller hospitals, understanding prescribing behaviors and tailoring ASP strategies to their unique practice settings is crucial to strengthening antimicrobial stewardship efforts.

## Methods

### Study design and setting

We conducted a single-center retrospective review of AU at a 151-bed safety-net community hospital in Chicago. The hospitalist service consists of one NP and one physician daily from 7am to 7 pm, with NPs and physicians managing patients independently on separate teams during daytime hours. A physician nocturnist provides coverage 7 pm to 7am without NP support. Our facility has a total of three hospitalists, four NPs, and 4 nocturnists.

### Antimicrobial stewardship program

Our facility has one ID physician, one lead ASP pharmacist, and four full time pharmacists without ID postgraduate training. Tools to support empiric antibiotic selection include syndrome-specific institutional guidelines informed by the facility’s antibiogram (Supplement 1). These resources are housed on the intranet and distributed during provider onboarding. Order sets based on these guidelines are also available.

### Data collection and outcome measures

Data gathered during the prospective audit and feedback (PAF) process for community acquired pneumonia (CAP), complicated intra-abdominal infections (cIAI), and urinary tract infections (UTI) from July 2022 to June 2023 was reviewed. Guideline concordance was defined as adherence to institutional empiric selection guidelines. Effectiveness was defined as empiric therapy providing≥80% local susceptibility based on our antibiogram. Guideline concordance, empiric selection effectiveness, and days of therapy per 1 000 patient days (DOT/1 000) were compared between NPs and physicians. Statistical comparisons for guideline discordance and antibiotic ineffectiveness were performed using the χ^2^ test. A *P* value of <.05 was considered statistically significant.

## Results

A total of 620 initial empiric antimicrobial selection events for CAP, cIAI and UTIs were documented in our PAF.

### Community-acquired pneumonia

**Figure 1.**
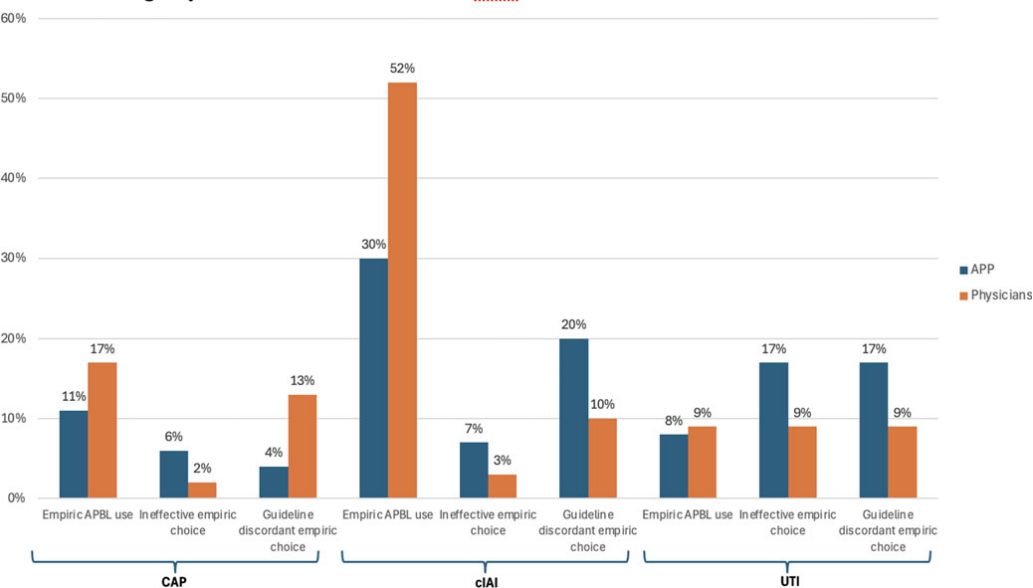
Empiric APBL use, ineffective empiric choices, and guideline-discordant empiric choices among physicians and nurse practitioners for community acquired pneumonia, complicated intra-abdominal infection, and urinary tract infection.

Of 236 CAP cases, 189 were managed by physicians and 47 by NPs. Guideline discordance was observed in 13% (24/189) of physicians and 4% (2/47) of NPs. Antibiotic ineffectiveness was 2% (4/189) for physicians and 6% (3/47) for NPs. Physicians managed a higher proportion of critically ill CAP patients (Figure 1, Supplement 2). Physicians were more likely to prescribe antipseudomonal beta-lactams (APBLs) for CAP compared to NPs (17% vs 7%).

### Complicated intra-abdominal infection

Of 175 cIAIs cases, 145 were managed by physicians and 30 from NPs. Guideline discordance was 20% (6/30) for NPs and 10% (14/145) for physicians. Antibiotic ineffectiveness was 3% (5/145) for physicians and 7% (2/30) for NPs. Physicians managed more cIAI with critical illness (Figure 1, Supplement 3). Physicians prescribed APBLs more frequently than NPs (52% vs 30%), while NPs prescribed quinolones (FQs) at a higher rate (33% vs 26%). Notably, 37% of patients prescribed FQs by NPs had beta-lactam allergies, compared to 22% of patients treated with FQs by physicians.

### Urinary tract infections

Of 209 UTI cases, 161 were managed by physicians and 48 by NPs. Guideline discordant and ineffective therapy was 9% (15/161) of physicians’ cases and 17% (8/48) of NPs’ cases. NPs were more likely to prescribe trimethoprim-sulfamethoxazole (TMP-SMX) (Figure 1, Supplement 4).

### Overall antibiotic utilization

**Figure 2.**
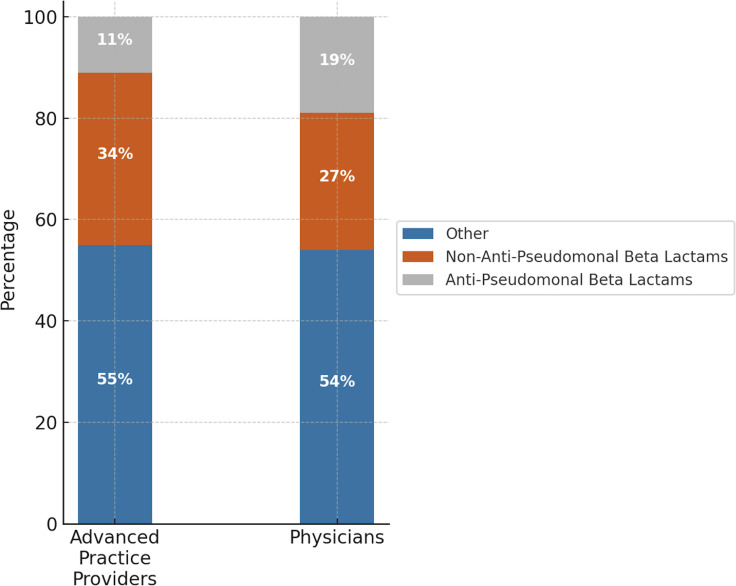
Empiric antimicrobial selection by physicians and nurse practitioners for community acquired pneumonia, complicated intra-abdominal infection, and urinary tract infection.

Total DOT/1 000 was 145. NPs accounted for 44 DOT/1 000, with 11% attributed to APBLs and 34% to non-APBLs. Physicians accounted for 21 DOT/1 000, with 19% APBL use and 27% non-APBL use (Figure 2). There was no statistically significant difference in guideline discordance between physicians and APPs (*P* = .61). However, antibiotic ineffectiveness was significantly more common among APPs (*P* = .03)

## Discussion

Prescribing differences between NPs and physicians highlight the need for targeted ASP education and interventions tailored by provider type. In our study, NPs adhered more to CAP guidelines than physicians, but had a higher rate of ineffective empiric choices. Physicians had higher APBL use, likely reflecting a higher proportion of critically ill patients. For cIAIs and UTIs, NPs demonstrated greater guideline non-adherence and higher rates of ineffective therapy based on local antibiograms. Guideline non-adherance was more than double that of physicians. NPs were also more likely to use non-preferred or ineffective agents based on our local antibiogram, such as fluoroquinolones for cIAIs or TMP-SMX for UTIs, which may reflect knowledge gaps. Discordance in cIAI and UTI may stem from use of agents with perceived gram-negative coverage but low local susceptibility. These findings highlight the need for targeted ASP education on antibiogram interpretation to optimize prescribing practices among NPs.

Variability in antimicrobial prescribing practices may be influenced by disparities in education and clinical training. Compared to physicians, NPs receive fewer formal education hours on antimicrobial therapy.^9^ While 95% of NP programs include antimicrobial lectures, most provide fewer than 10 total hours of instruction.^9^ In a survey across five hospitals, NPs reported lower confidence in de-escalating antimicrobials and were less likely to select empiric therapy based on an antibiogram.^10^ Disparities in training may contribute to differences in prescribing patterns and reinforce the need for structured ASP interventions tailored to NPs.

Our study has several limitations. As a single-center study with a small sample size, findings may not be generalizable. The impact of overnight moonlighters, who may have limited engagement with local prescribing protocols, was not specifically evaluated. Additionally, outcomes related to prescribing differences were not evaluated, limiting insight into the effects of guideline non-adherence. Despite these limitations, our study identifies key prescribing differences between NPs and physicians, underscoring the need for tailored educational strategies and ASP interventions.

## Supporting information

10.1017/ash.2025.10058.sm001 Balaji et al. supplementary material Balaji et al. supplementary material

## Supplementary material

The supplementary material for this article can be found at https://doi.org/10.1017/ash.2025.10058.
