# Supplementary material for: A comparative study of antimicrobial prescribing practices for common infectious syndromes among physicians and nurse practitioners in a safety-net hospital
Source: Antimicrob Steward Healthc Epidemiol. 2025 Jun 30;5(1):e140. doi: 10.1017/ash.2025.10058 (PMC12224140; doi:10.1017/ash.2025.10058)

**SUPPLEMENTS**

**Supplement 1.** Institutional guidelines for empiric antimicrobial selection based on syndrome and severity

| Syndrome | How to define it | Severity | Initial Treatment |
| --- | --- | --- | --- |
| Community acquired pneumonia  (CAP) | PNA without hospital, NH, LTACH contact in past 90 days | Leukocytosis, need for ICU care or respiratory support | **Mild/Moderate**  Ceftriaxone PLUS Azithromycin  Levofloxacin for PCN allergic  **Severe**  Piperacillin-tazo PLUS Azithromycin  Adding vancomycin to mild-mod-severe is acceptable if risk factors or concern for MRSA  Aztreonam for PCN allergic |
| Intra-abdominal Infection  (cIAI) | Includes peritonitis, cholangitis, liver abscess, abdominal abscess, diverticular abscess, diverticulitis.  Does not include diarrhea. | Leukocytosis, fever, hemodynamic instability.  If bacteremia is present and patient is critically ill, broad agents are acceptable. | **Mild/Moderate**  Ceftriaxone PLUS Metronidazole  **Severe illness**  Piperacillin-tazobactam OR meropenem  IF Cefepime always ADD metronidazole  IF Aztreonam always ADD metronidazole AND vancomycin  **Other comments**  Ciprofloxacin or Levofloxacin plus metronidazole is not recommended for initial management.  Diarrhea is not considered an IAB. Antibiotics are not indicated for diarrhea. |
| Urinary tract infection  OR  Pyelonephritis  (UTI) | Urinary symptoms or radiographic image with renal infection. | Leukocytosis, fever, hemodynamic instability.  If bacteremia is present and patient is critically ill, broad agents are acceptable. | **Moderate**  Ceftriaxone  **Severe**  Piperacillin OR meropenem, only for critically ill or history of drug resistance  Aztreonam is alternative for allergic patients |

**Supplement 2.** CAP severity and empiric antimicrobial selection by physicians and APPs


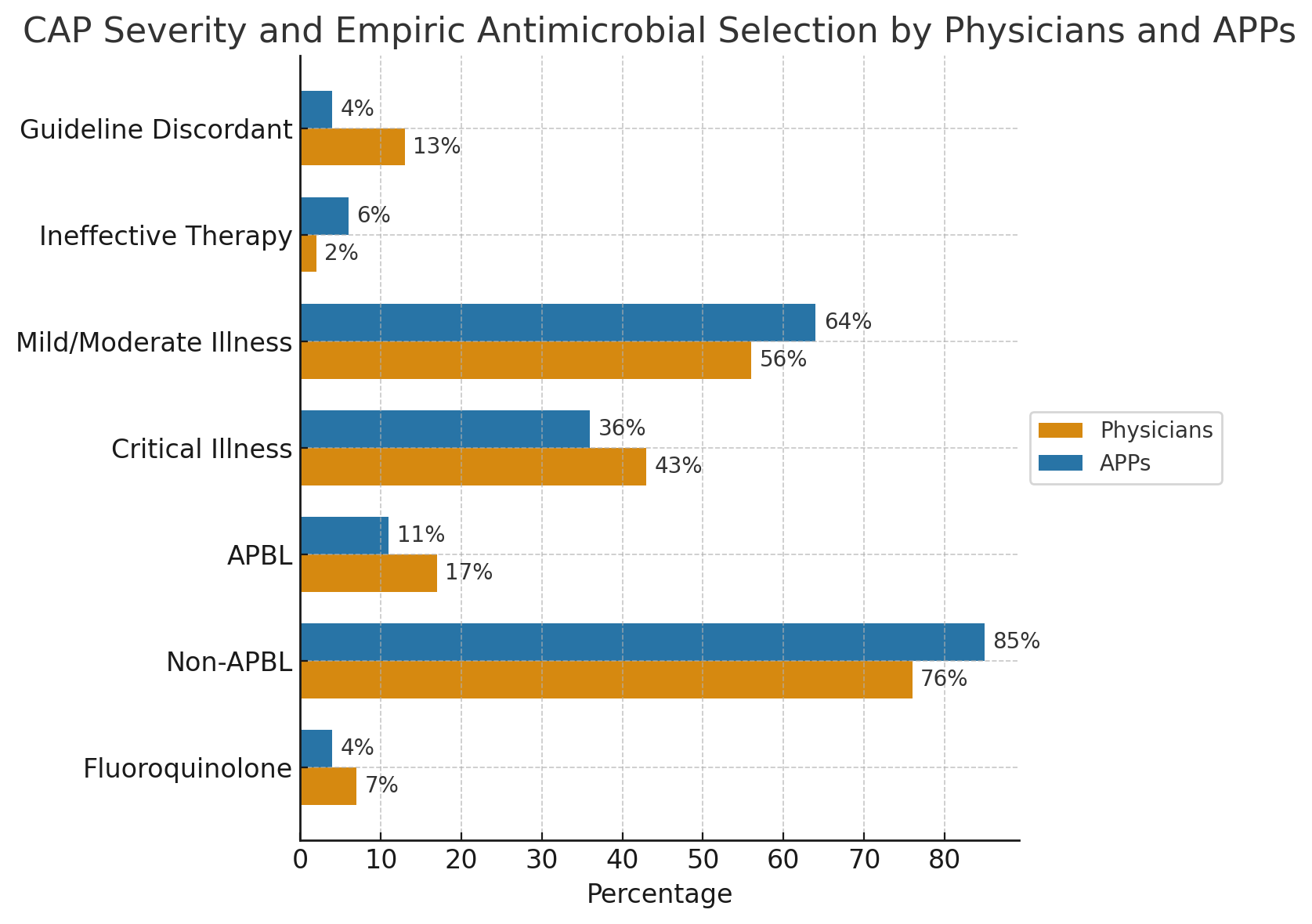


**Supplement 3.** cIAI severity and empiric antimicrobial selection by physicians and APPs


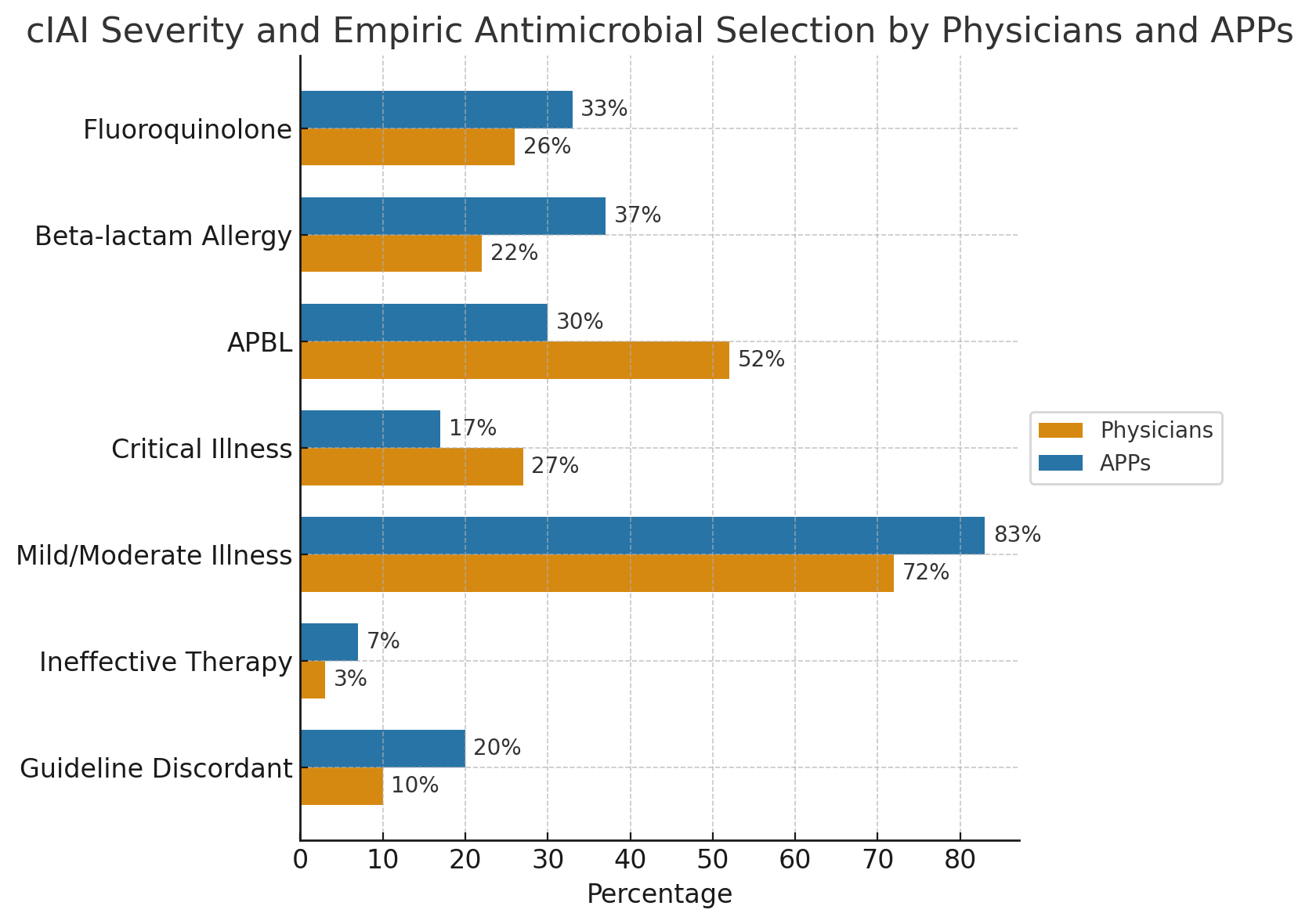


**Supplement 4.** UTI severity and empiric antimicrobial selection by physicians and APPs


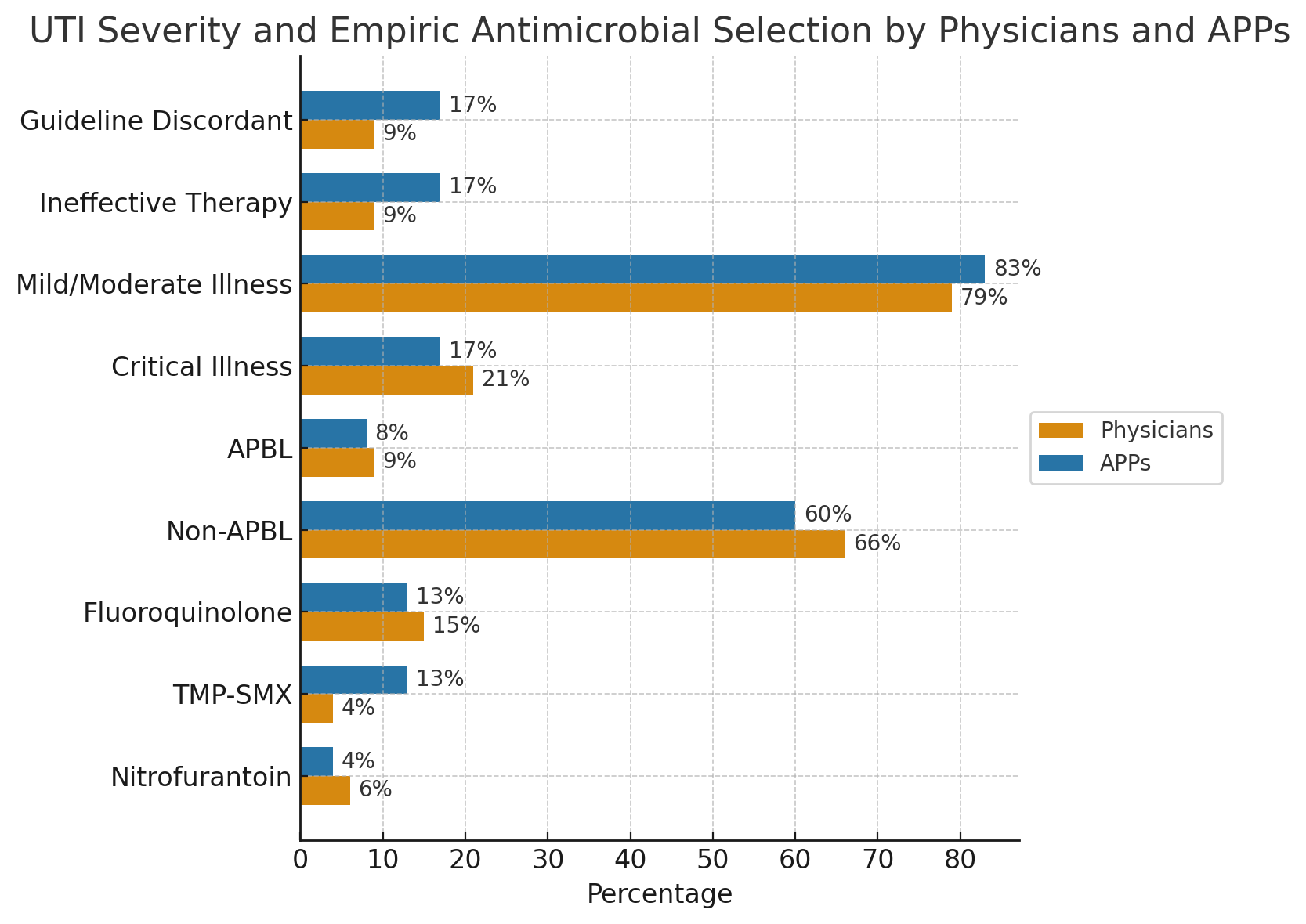

Supplement: Balaji et al. supplementary material [file S2732494X25100582sup001.docx]
